# Recent Findings on the Role of Gelatinases (Matrix Metalloproteinase-2 and -9) in Osteoarthritis

**DOI:** 10.1155/2012/834208

**Published:** 2012-07-26

**Authors:** Olimpio Galasso, Filippo Familiari, Marco De Gori, Giorgio Gasparini

**Affiliations:** Department of Orthopaedic and Trauma Surgery, School of Medicine, University Magna Græcia of Catanzaro, Europa Avenue, 88100 Catanzaro, Italy

## Abstract

Several studies dealing with the pathomechanisms of OA refer to MMP-1, -3, -7, -8, and -13 whereas a smaller number of investigations have pointed out the pathogenic role of gelatinases in OA. These gelatinases are best known for their involvement in pulmonary, myocardial, and neoplastic disease but they are emerging as important proteases implicated in the OA progression. This paper highlights the role of the gelatinases as emerging factors in OA pathogenesis through the regulation of subchondral bone resorption and microvascular invasion. The most significant new findings over the last year that add to our knowledge of the activity of these proteins in OA have been reported.

## 1. Introduction

Hereditary, mechanical, and biological factors participate in the causation of osteoarthritis (OA) that is finally characterized by a net loss of the articular cartilage, resulting in pain, deformity, loss of motion, and decreased function [1]. Changes in the normal homeostasis of articular cartilage and subchondral bone during OA are caused by the combination of (1) chondrocyte death, (2) increased degradation, and (3) decreased production of extracellular matrix (ECM).

The matrix metalloproteinases (MMPs) are a family of Zn2+-dependent endopeptidases that regulate the degradation of ECM and play a pivotal role in many physiological and pathological processes of different tissues [2–4]. Indeed, the timely breakdown of ECM is essential for embryonic development, morphogenesis, reproduction, and tissue resorption and remodeling [5].

MMPs are categorized into the following groups: collagenases (MMP-1, MMP-8, and MMP-13), gelatinases (MMP-2 and MMP-9), stromelysins (MMP-3, MMP-10, and MMP-11), matrilysin (MMP-7), metalloelastase (MMP-12), and membrane-type matrix metalloproteinases (MT-MMP 1, 2, 3, and 4) [6].

Several studies dealing with the pathomechanisms of OA refer to MMP-1, -3, -7, -8, and -13 [7–12] whereas a smaller number of investigations have pointed out the pathogenic role of gelatinases in OA. Indeed, the ECM of articular cartilage is primarily composed of type II collagen and aggrecan that are not the primary substrates of gelatinases. These gelatinases are best known for their involvement in pulmonary [13], myocardial [14], and neoplastic disease [15] but they are emerging as important proteases implicated in the OA progression.

In this paper, we summarize the present state of knowledge of gelatinases' role in OA. The most significant new findings over the last year that add to our knowledge of the activity of these proteins in OA have been reported. As far as we know, no previous articles to comprehensively cover this topic are available in the literature.

## 2. Materials and Methods

We performed a literature search of the MEDLINE/PubMed, Excerpta Medica/EMBASE databases for articles published during the past 30 years (1981–2011). Our purpose was to identify all English-language literature included under the key-words *metalloproteinase-2, metalloproteinase-9, MMP-2, MMP-9, gelatinase A, and gelatinase B *alone or combined with *osteoarthritis. *The contents of 166 pertinent abstracts or full-text articles were identified during our literature search. Then, abstracts, case reports, and letters to the editor were excluded thus leaving 101 articles to be finally considered for this paper.

We have cited articles that meet accepted quality standards for design and reporting [16]. Review articles and book chapters are also cited to provide readers with more details and references. No attempt was made to solicit unpublished data or to retrieve additional information from any of the authors of the studies.

## 3. Structure and Function of Gelatinases

MMP-2 (gelatinase A, 72 kDa type IV collagenase) is a matrix metalloproteinase which was first described and purified from highly metastatic murine tumors [17, 18] and cultured human melanoma cells [19]. MMP-2 is abundantly expressed in fibroblasts, endothelial, and epithelial cells [20–22] and it is secreted as proenzyme and activated at the cell surface. Its activation is mediated by the membrane-type metalloproteinase-1 (MT-MMP 1) [23, 24]. MMP-2 activation involves tissue inhibitor of MMP (TIMP)-2 as a bridging molecule between MT-MMP 1 and pro-MMP-2. Thus, net activity of MT-MMP 1 and MMP-2 depends on TIMP-2 concentration [25].

MMP-2 participates in ECM degradation with a wide range of substrates. Indeed, it is able to degrade type I, IV, V, VII, and X collagens, laminin, elastin, fibronectin, and proteoglycans [26–28]. Normal adult articular chondrocytes express significant amount of MMP-2 both *in vivo *and *in vitro *suggesting this metalloproteinase is involved in physiological collagen turnover of human adult articular cartilage [29].

MMP-9 (gelatinase B, 92 kDa type IV collagenase) was first purified from human macrophages [20]. Its expression is limited to osteoclasts, macrophages, trophoblasts, hippocampal neurocytes, and migrating keratinocytes [22]. In particular MMP-9 and cathepsin K are considered the most abundant proteases in osteoclasts [30]. MMP-9 is controlled by growth factors, chemokines, and other stimulatory signals [31]. It is secreted as an inactive precursor form named pro-MMP-9 that forms a tight complex with TIMP-1 and TIMP-3. The complex of pro-MMP-9 and TIMP-1 [24, 32], the plasmin, and the complex plasminogen/MMP-3 are activators of pro-MMP-9 [26, 33].

Gelatinase B has been shown to dissolve extracellular matrix, initiating and promoting new vessel formation [34]. Furthermore, this enzyme is known to cleave native type IV, V, VII, and X collagens and elastin, as well as the products of collagens types I, II, and III after proteolysis by collagenases (Table 1) [3].

*In vivo *the MMP-9 is poorly expressed in normal adult chondrocytes suggesting that this gelatinase is hardly involved in physiological collagen turnover [35].

## 4. Gelatinases in OA

**Figure 1:**
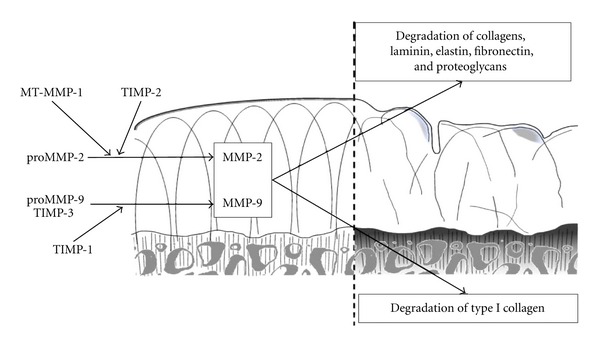
The activation of gelatinases and their activity during osteoarthritis. Normal (left) and osteoarthritic (right) cartilage and subchondral bone.

It was demonstrated that the expression of both MMP-2 and MMP-9 is enhanced in osteoarthritic cartilage (Figure 1) [36]. Also the MT-MMP 1, which activates the MMP-2, was found highly expressed in the chondrocytes during OA [37]. Duerr et al.  evaluated the quantitative expression levels and the distribution of MMP-2 and MMP-9 both in normal and osteoarthritic cartilage and in cultured articular chondrocytes [29, 35]. They found that in osteoarthritic cartilage degradation, MMP-9 is expressed at a very much lower level than MMP-2. Accordingly, Wang et al. reported minimal changes in the cartilage expression of MMP-9 in an experimental model of secondary OA [38]. Indeed, this study showed that the experimentally induced cartilage damage led to OA-like lesions with disarrangement of cellular disposition, cell-free areas, coagulation necrosis, pyknotic nuclei, and local loss of extracellular matrix accompanied by absent immunopositive expression of MMP-3, MMP-9, TIMP-1, and aggrecan.

Current data suggest that during OA, the activity of gelatinases is higher on the subchondral bone rather than on cartilage ECM [39]. Indeed, MMP-2 is capable of cleaving type I and other fibrillar collagens [40] that are uncommon in the ECM of articular cartilage but are present in the ECM of subchondral bone. Using a specific gelatinase inhibitor, Hill et al.  showed that both the gelatinases participate in the degradation of the organic matrix of bone [41]. Mansell and Bailey investigating the cancellous bone metabolism during OA, reported an increased potential for collagen degradation in presence of increased levels of both pro- and active MMP-2 [42]. What is evident from this study is that OA cancellous bone is metabolically active compared with normal tissue. Such differences in turnover might result in altered joint morphology, which in turn might exacerbate the osteoarthritic process (Figure 1). Osteoclasts constitutively express MMP-2, and synthesize MMP-9, MMP-3, and TIMP-1 in response to IL-1*α* stimulation, and during OA the increased levels of osteoclast-derived MMPs might contribute to osteoclast lacunar resorption [43]. This hypothesis concurs with the demonstration of higher plasma levels of MMP-9 in patients with rapidly destructive hip OA in comparison patients with OA or normal controls [44]. The higher detected amount of MMP-9 could be explained by the wide number of osteoclasts, which are one source of MMP-9, observed around necrotic bone in subchondral areas in rapidly destructive hip OA [45, 46]. Nor should be excluded the enhanced production of MMP-9 by synovial cells of patients with this kind of OA [47]. A direct route into the bloodstream via the subchondral microcirculatory system and an indirect route from synovial fluid into circulation could explain the higher plasma levels of MMP-9 in OA [48].

The work of Bellido et al.  strengthens the role of the subchondral bone as a key player in the puzzle of OA development [49]. They found raised subchondral MMP-9 levels in patient suffering from OA demonstrating a clear increase in local bone resorption with a decreased thickness of the subchondral plate. The subchondral plate that separates articular noncalcified cartilage from the bone marrow cavity consists of calcified cartilage and subchondral lamellar bone layers [50]. Any impairment in subchondral bone quality makes this organ not able to receive and properly distribute loads from and/or to the articular cartilage. Thus, changes at subchondral bone may aggravate cartilage damage. Indeed, the authors observed a direct correlation between subchondral structural parameters and cartilage damage evaluated with the Mankin's scale. The presence of subchondral bone resorption pits composed by MMP-producing cells derived from bone marrow has been previously evidenced together with their contribution to cartilage degradation through the invasion of this tissue [51].

The type II collagen, the most abundant collagen expressed in articular cartilage, is natively degraded by MMP-1, -8, -13, and -14 producing fragments. However, denatured and partially degraded collagen II is further degraded by gelatinases and stromelysins thus obtaining a C-terminal peptide fragment, named CTX-II. This fragment is used as an urinary marker of cartilage degradation because it was found to correlate with cartilage loss in animal models of OA [52] and increased CTX-II levels in patients with OA compared with controls were reported [53]. Correlations of CTX-II with clinical assessment [54] and X-rays [55] or MRI [56, 57] evaluation of human OA have been found.

## 5. Recent Findings

Recent articles confirm that the gelatinases influence OA onset and progression regulating the subchondral bone remodeling (Table 2). In particular, a predominant role of MMP-9 emerged during, last year. Among various MMPs, the total MMP-9 level is positively correlated with the total MMP-13 levelin OA [58], and it has been hypothesized that this gelatinase might be involved in the activation of pro-MMP-13 through yet unknown mechanisms. Notably, MMP-13 has long been considered as the major enzyme involved in cartilage erosion during OA, thus MMP-9 might play a role, at least cooperatively, in joint degradation.

High levels of VEGF and gelatinases were confirmed in osteoarthritic fluid but MMP-2 and -9 levels were not significantly associated with VEGF expression [59]. Differently, it was previously demonstrated that the MMP-9 levels correlated with synovial fluid VEGF levels and with the pattern of vascularity found in the synovial membrane tissue of patients with arthritis. Moreover, synovial membrane explants stimulated with VEGF increased supernatant MMP-9 levels by 2-fold from baseline [60]. Recently, Li et al. [61] demonstrated that higher MMP-9 expression was found in case of severe OA in comparison with early OA and this expression correlated in a direct manner with vascular invasion. These findings together suggest a possible relationship among gelatinases and the angiogenesis noted with OA development, and it is tempting to speculate that MMP-9 may be therapeutic target for angiogenesis inhibition.

New regulatory mechanisms of gelatinase expression have been proposed. The increase of gelatinases in OA might be induced by abnormal mechanical pressure applied to the articulation. Indeed, cyclic compression on osteoblasts from osteoarthritic subchondral bone increases the expression of genes coding for MMP-9. Conversely, MMP-2 was not modulated by compression, suggesting that this is not a mechanosensitive gene [62]. An interesting article demonstrated that TGF-*β*1 protects articular cartilage by downregulating the expression of MMP-9 of chondrocytes and synoviocytes in OA, which may delay the biological behavior of this disease. The authors found a negative correlation between the expression of MMP-9 protein and TGF-*β*1 protein, and between the expression of MMP-9 mRNA and TGF-*β*1 mRNA in the specimens of osteoarthritic cartilage [63].

Based on the demonstration that high CTX-II levels are predictive of OA progression [53–56], De Ceuninck et al. [64] recently highlighted the role of this degradation fragment of gelatinases not only as a diagnostic, but also as a prognostic reliable biomarker of OA. Conversely, Kim et al. [58] addressed the role of MMP-9 as possible biomarker for differentiating between OA and other articular cartilage diseases.

## 6. Conclusions

Collagenases have long been considered as the major enzymes involved in OA occurrence and progression. This paper highlights the role of the gelatinases as important factors in OA pathogenesis through the regulation of subchondral bone resorption. New intriguing regulatory mechanisms of gelatinases expression and further data about the relationship between these proteins and microvascular invasion commonly found in OA have been demonstrated over the last year. Experimental strategies that modify the expression and/or the activity of MMPs might consider the gelatinases as promising targets for the treatment of OA disease.

## Figures and Tables

**Table 1:** Metalloproteinase-2 and metalloproteinase-9 chromosome location, biological effects and substrates of action.

Gene	Biological effects	Main substrates
*MMP-2 * 16q13-q21	(1) Adipocyte migration (2) Apoptosis (amnion epithelial cells) (3) Conversion of vasodilator to vasoconstrictor (4) ECM degradation (5) Enhanced collagen affinity (6) Epithelial cell migration (7) Generation of vasoconstrictor (8) Increased bioavailability of IGF1 and cell proliferation (9) Increased bioavailability of TGF-*β* (10) Mesenchymal cell differentiation with inflammatory phenotype (11) Neural apoptosis leading to neurodegeneration (12) Neurite outgrowth	(13) Aggrecan
		(14) Collagen type I
		(15) Collagen type III
		(16) Collagen type IV
		(17) Collagen type VII
		(18) Collagen type X
		(19) Collagen type XI
		(20) Decorin
		(21) Elastin
		(22) Fibrinogen
		(23) Gelatin
		(24) Laminin
		(25) Plasminogen
		(26) proMMP-9
		(27) proMMP-13
		(28) Vitronectin

*MMP-9 * 20q11.2-q13.1	(29) Bioavailability of TGF-*β* (30) Generation of angiostatin-like fragment (31) ECM degradation (32) Enhanced collagen affinity (33) Hypertrophic chondrocytes apoptosis and recruitment of osteoclast (34) Pro-inflammatory (35) Reduced IL-2 response (36) Tumor cell resistance (37) Thymic neovascularization	(38) Collagen type I
		(39) Collagen type IV
		(40) Collagen type VII
		(41) Collagen type X
		(42) Collagen type XI
		(43) Collagen type XVIII
		(44) Elastin
		(45) Fibronectin
		(46) Gelatin
		(47) Laminin
		(48) proMMP-2
		(49) proMMP-9
		(50) Vitronectin

**Table 2:** Articles dealing with the gelatinases over the last year.

Authors	Type of study	Main results
De Ceunink et al. [64]	Review	MMP-2 and MMP-9 useful as OA biomarkers
Kim et al. [58]	Human	MMP-9 is involved in activation of MMP-13
Kim et al. [59]	Human	MMP-9 is associated with VEGF expression in OA
Li et al. [61]	Human	MMP-9 expression correlates with vascular invasion in severe OA
Sanchez et al. [62]	Human	The expression of MMP-9 increases by cyclic compression on osteoblasts from osteoarthritic subchondral bone
Guo et al. [63]	Human	TGF-*β*1 protects articular cartilage downregulating

## References

[1] Tanaka S, Hamanishi C, Kikuchi H, Fukuda K (1998). Factors related to degradation of articular cartilage in osteoarthritis: a review. *Seminars in Arthritis and Rheumatism*.

[2] Shapiro SD (1999). Diverse roles of macrophage matrix metalloproteinases in tissue destruction and tumor growth. *Thrombosis and Haemostasis*.

[3] Jones CB, Sane DC, Herrington DM (2003). Matrix metalloproteinases: a review of their structure and role in acute coronary syndrome. *Cardiovascular Research*.

[4] Creemers EE, Cleutjens JP, Smits JF, Daemen MJ (2001). Matrix metalloproteinase inhibition after myocardial infarction: a new approach to prevent heart failure?. *Circulation Research*.

[5] Gronski TJ, Martin RL, Kobayashi DK (1997). Hydrolysis of a broad spectrum of extracellular matrix proteins by human macrophage elastase. *The Journal of Biological Chemistry*.

[6] Sapolsky AI, Howell DS (1982). Further characterization of a neutral metalloprotease isolated from human articular cartilage. *Arthritis and Rheumatism*.

[7] Galloway WA, Murphy G, Sandy JD, Gavrilovic J, Cawtson TE, Reynolds JJ (1983). Purification and characterization of a rabbit bone metalloproteinase that degrades proteoglycan and other connective-tissue components. *The Biochemical Journal*.

[8] Gunja-Smith Z, Nagase H, Woessner JF (1989). Purification of the neutral proteoglycan-degrading metalloproteinase from human articular cartilage tissue and its identification as stromelysin matrix metalloproteinase-3. *The Biochemical Journal*.

[9] Fosang AJ, Neame PJ, Hardingham TE, Murphy G, Hamilton JA (1991). Cleavage of cartilage proteoglycan between G1 and G2 domains by stromelysins. *The Journal of Biological Chemistry*.

[10] Fosang AJ, Neame PJ, Last K, Hardingham TE, Murphy G, Hamilton JA (1992). The interglobular domain of cartilage aggrecan is cleaved by PUMP, gelatinases, and cathepsin B. *The Journal of Biological Chemistry*.

[11] Fosang AJ, Last K, Knauper V (1993). Fibroblast and neutrophil collagenases cleave at two sites in the cartilage aggrecan interglobular domain. *The Biochemical Journal*.

[12] Fosang AJ, Last K, Knäuper V, Murphy G, Neame PJ (1996). Degradation of cartilage aggrecan by collagenase-3 (MMP-13). *FEBS Letters*.

[13] Chakrabarti S, Patel KD (2005). Matrix metalloproteinase-2 (MMP-2) and MMP-9 in pulmonary pathology. *Experimental Lung Research*.

[14] Kandasamy AD, Chow AK, Ali MA, Schulz R (2010). Matrix metalloproteinase-2 and myocardial oxidative stress injury: beyond the matrix. *Cardiovascular Research*.

[15] Turpeenniemi-Hujanen T (2005). Gelatinases (MMP-2 and -9) and their natural inhibitors as prognostic indicators in solid cancers. *Biochimie*.

[16] Gasparyan AY, Ayvazyan L, Blackmore H, Kitas GD (2011). Writing a narrative biomedical review: considerations for authors, peer reviewers, and editors. *Rheumatology International*.

[17] Liotta LA, Abe S, Robey PG, Martin GR (1979). Preferential digestion of basement membrane collagen by an enzyme derived from a metastatic murine tumor. *Proceedings of the National Academy of Sciences of the United States of America*.

[18] Salo T, Liotta LA, Tryggvason K (1983). Purification and characterization of a murine basement membrane collagen-degrading enzyme secreted by metastatic tumor cells. *The Journal of Biological Chemistry*.

[19] Hoyhtya M, Hujanen E, Turpeenniemi-Hujanen T, Thorgeirsson U, Liotta LA, Tryggvason K (1990). Modulation of type-IV collagenase activity and invasive behavior of metastatic human melanoma (A2058) cells in vitro by monoclonal antibodies to type-IV collagenase. *International Journal of Cancer*.

[20] Vartio T, Hovi T, Vaheri A (1982). Human macrophages synthesize and secrete a major 95,000-dalton gelatin-binding protein distinct from fibronectin. *The Journal of Biological Chemistry*.

[21] Salo T, Oikarinen J (1985). Regulation of type IV collagen degrading enzyme by cortisol during human skin fibroblast growth. *Biochemical and Biophysical Research Communications*.

[22] Hipps DS, Hembry RM, Docherty AJ, Reynolds JJ, Murphy G (1991). Purification and characterization of human 72-kDa gelatinase (type IV collagenase). Use of immunolocalisation to demonstrate the non-coordinate regulation of the 72-kDa and 95-kDa gelatinases by human fibroblasts.. *Biological Chemistry Hoppe-Seyler*.

[23] Strongin AY, Collier I, Bannikov G, Marmer BL, Grant G, Goldberg G (1995). Mechanism of cell surface activation of 72-kDa type IV collagenase. *The Journal of Biological Chemistry*.

[24] Visse R, Nagase H (2003). Matrix metalloproteinases and tissue inhibitors of metalloproteinases: structure, function, and biochemistry. *Circulation Research*.

[25] Sato H, Takino T (2010). Coordinate action of membrane-type matrix metalloproteinase-1 (MT1-MMP) and MMP-2 enhances pericellular proteolysis and invasion. *Cancer Science*.

[26] Ramos-De Simone N, Hahn-Dantona E, Sipley J, Nagase H, French DL, Quigley JP (1999). Activation of matrix metalloproteinase-9 (MMP-9) via a converging plasmin/stromelysin-1 cascade enhances tumor cell invasion. *The Journal of Biological Chemistry*.

[27] Woessner JF (1999). Matrix metalloproteinases. *The Journal of Biological Chemistry*.

[28] Sternlicht MD, Werb Z (2001). How matrix metalloproteinases regulate cell behavior. *Annual Review of Cell and Developmental Biology*.

[29] Duerr S, Stremme S, Soeder S, Bau B, Aigner T (2004). MMP-2/gelatinase A is a gene product of human adult articular chondrocytes and is increased in osteoarthritic cartilage. *Clinical and Experimental Rheumatology*.

[30] Logar DB, Komadina R, Preželj J, Ostanek B, Trošt Z, Marc J (2007). Expression of bone resorption genes in osteoarthritis and in osteoporosis. *Journal of Bone and Mineral Metabolism*.

[31] Fini ME, Cook JR, Mohan R, Brinckerhoff CE, Park WC, Mecham RP (1998). Regulation of matrix metalloproteinase gene expression. *Matrix Metalloproteinases*.

[32] Nagase H, Visse R, Murphy G (2006). Structure and function of matrix metalloproteinases and TIMPs. *Cardiovascular Research*.

[33] Mazzieri R, Masiero L, Zanetta L (1997). Control of type IV collagenase activity by the urokinase-plasmin system: a regulatory mechanism with cell-bound reactants. *The EMBO Journal*.

[34] Sang QX (1998). Complex role of matrix metalloproteinases in angiogenesis. *Cell Research*.

[35] Söder S, Roach HI, Oehler S, Bau B, Haag J, Aigner T (2006). MMP-9/gelatinase B is a gene product of human adult articular chondrocytes and increased in osteoarthritic cartilage. *Clinical and Experimental Rheumatology*.

[36] Mohtai M, Smith RL, Schurman DJ (1993). Expression of 92-kD type IV collagenase/gelatinase (gelatinase B) in osteoarthritic cartilage and its induction in normal human articular cartilage by interleukin 1. *The Journal of Clinical Investigation*.

[37] Kinoshita T, Sato H, Okada A (1998). TIMP-2 promotes activation of progelatinase A by membrane-type 1 matrix metalloproteinase immobilized on agarose beads. *The Journal of Biological Chemistry*.

[38] Wang GW, Wang MQ, Wang XY, Yu SB, Liu XD, Jiao K (2010). Changes in the expression of MMP-3, MMP-9, TIMP-1 and aggrecan in the condylar cartilage of rats induced by experimentally created disordered occlusion. *Archives of Oral Biology*.

[39] Hulejová H, Barešová V, Klézl Z, Polanská M, Adam M, Šenolt L (2007). Increased level of cytokines and matrix metalloproteinases in osteoarthritic subchondral bone. *Cytokine*.

[40] Aimes RT, Quigley JP (1995). Matrix metalloproteinase-2 is an interstitial collagenase. Inhibitor-free enzyme catalyzes the cleavage of collagen fibrils and soluble native type I collagen generating the specific 3/4- and 1/4-length fragments. *The Journal of Biological Chemistry*.

[41] Hill PA, Docherty AJ, Bottomley KM (1995). Inhibition of bane resorption in vitro by selective inhibitors of gelatinase and collagenase. *The Biochemical Journal*.

[42] Mansell JP, Bailey AJ (1998). Abnormal cancellous bone collagen metabolism in osteoarthritis. *The Journal of Clinical Investigation*.

[43] Seibel MJ, Duncan A, Robins SP (1989). Urinary hydroxy-pyridinium crosslinks provide indices of cartilage and bone involvement in arthritic diseases. *The Journal of Rheumatology*.

[44] Masuhara K, Nakai T, Yamaguchi K, Yamasaki S, Sasaguri Y (2002). Significant increases in serum and plasma concentrations of matrix metalloproteinases 3 and 9 in patients with rapidly destructive osteoarthritis of the hip. *Arthritis & Rheumatism*.

[45] Mitrovic DR, Riera H (1992). Synovial, articular cartilage and bone changes in rapidly destructive arthropathy (osteoarthritis) of the hip. *Rheumatology International*.

[46] Buisson-Legendre N, Smith S, March L, Jackson C (2004). Elevation of activated protein C in synovial joints in rheumatoid arthritis and its correlation with matrix metalloproteinase 2. *Arthritis & Rheumatism*.

[47] Masuhara K, Lee SB, Nakai T, Sugano N, Ochi T, Sasaguri Y (2000). Matrix metalloproteinases in patients with osteoarthritis of the hip. *International Orthopaedics*.

[48] Naito K, Takahashi M, Kushida K (1999). Measurement of matrix metalloproteinases (MMPs) and tissue inhibitor of metalloproteinases-1 (TIMP-1) in patients with knee osteoarthritis: comparison with generalized osteoarthritis. *Rheumatology*.

[49] Bellido M, Lugo L, Roman-Blas JA (2010). Subchondral bone microstructural damage by increased remodelling aggravates experimental osteoarthritis preceded by osteoporosis. *Arthritis Research and Therapy*.

[50] Duncan H, Jundt J, Riddle JM, Pitchford W, Christopherson T (1987). The tibial subchondral plate. A scanning electron microscopic study. *Journal of Bone and Joint Surgery A*.

[51] Shibakawa A, Yudoh K, Masuko-Hongo K, Kato T, Nishioka K, Nakamura H (2005). The role of subchondral bone resorption pits in osteoarthritis: MMP production by cells derived from bone marrow. *Osteoarthritis and Cartilage*.

[52] Duclos ME, Roualdes O, Cararo R, Rousseau JC, Roger T, Hartmann DJ (2010). Significance of the serum CTX-II level in an osteoarthritis animal model: a 5-month longitudinal study. *Osteoarthritis and Cartilage*.

[53] Sharif F, Kirwan J, Charni N, Sandell LJ, Whittles C, Garnero P (2007). A 5-yr longitudinal study of type IIA collagen synthesis and total type II collagen degradation in patients with knee osteoarthritis—association with disease progression. *Rheumatology*.

[54] Jung M, Christgau S, Lukoschek M, Henriksen D, Richter W (2004). Increased urinary concentration of collagen type II C-telopeptide fragments in patients with osteoarthritis. *Pathobiology*.

[55] Sowers MF, Karvonen-Gutierrez CA, Yosef M (2009). Longitudinal changes of serum COMP and urinary CTX-II predict X-ray defined knee osteoarthritis severity and stiffness in women. *Osteoarthritis and Cartilage*.

[56] Byrjalsen I, Karsdal MA, Qvist P, Christiansen C (2009). Increased urinary excretion of C-telopeptides of type II collagen (CTX-II) predicts cartilage loss over 21 months by MRI. *Osteoarthritis and Cartilage*.

[57] Dam EB, Loog M, Christiansen C (2009). Identification of progressors in osteoarthritis by combining biochemical and MRI-based markers. *Arthritis Research and Therapy*.

[58] Kim KS, Lee YA, Choi HM, Yoo MC, Yang HI Implication of MMP-9 and urokinase plasminogen activator (uPA) in the activation of pro-matrix metalloproteinase (MMP)-13.

[59] Kim KS, Choi HM, Lee YA (2011). Expression levels and association of gelatinases MMP-2 and MMP-9 and collagenases MMP-1 and MMP-13 with VEGF in synovial fluid of patients with arthritis. *Rheumatology International*.

[60] Fraser A, Fearon U, Reece R, Emery P, Veale DJ (2001). Matrix metalloproteinase 9, apoptosis, and vascular morphology in early arthritis. *Arthritis and Rheumatism*.

[61] Li H, Miao SB, Dong LH (2012). Clinicopathological correlation of Krüppel-like factor 5 and matrix metalloproteinase-9 expression and cartilage degeneration in human osteoarthritis. *Pathology, Research and Practice*.

[62] Sanchez C, Pesesse L, Gabay O (2012). Regulation of subchondral bone osteoblast metabolism by cyclic compression. *Arthritis Rheumatism*.

[63] Guo J, Zhang W, Li Q, Gan H, Wang Z (2011). Significance of expressions of matrix metalloproteinase 9 mRNA, transforming growth factor beta1, mRNA and corresponding proteins in osteoarthritis. *Chinese Journal of Reparative and Reconstructive Surgery*.

[64] De Ceuninck F, Sabatini M, Pastoureau P (2011). Recent progress toward biomarker identification in osteoarthritis. *Drug Discovery Today*.

